# Crack Propagation Velocity Determination by High-speed Camera Image Sequence Processing

**DOI:** 10.3390/ma13194415

**Published:** 2020-10-03

**Authors:** Frank Liebold, Ali A. Heravi, Oliver Mosig, Manfred Curbach, Viktor Mechtcherine, Hans-Gerd Maas

**Affiliations:** 1 Institute of Photogrammetry and Remote Sensing, TU Dresden, 01062 Dresden, Germany; hans-gerd.maas@tu-dresden.de; 2 Institute of Construction Materials, TU Dresden, 01062 Dresden, Germany; ali.a.heravi@tu-dresden.de (A.A.H.); Mechtcherine@tu-dresden.de (V.M.); 3 Institute of Concrete Structures, TU Dresden, 01062 Dresden, Germany; oliver.mosig@tu-dresden.de (O.M.); manfred.curbach@tu-dresden.de (M.C.)

**Keywords:** high-speed camera, crack propagation velocity, image sequence analysis, crack analysis, material testing, deformation measurement

## Abstract

The determination of crack propagation velocities can provide valuable information for a better understanding of damage processes of concrete. The spatio-temporal analysis of crack patterns developing at a speed of several hundred meters per second is a rather challenging task. In the paper, a photogrammetric procedure for the determination of crack propagation velocities in concrete specimens using high-speed camera image sequences is presented. A cascaded image sequence processing which starts with the computation of displacement vector fields for a dense pattern of points on the specimen’s surface between consecutive time steps of the image sequence chain has been developed. These surface points are triangulated into a mesh, and as representations of cracks, discontinuities in the displacement vector fields are found by a deformation analysis applied to all triangles of the mesh. Connected components of the deformed triangles are computed using region-growing techniques. Then, the crack tips are determined using the principal component analysis. The tips are tracked in the image sequence and the velocities between the time stamps of the images are derived. A major advantage of this method as compared to the established techniques is in the fact that it allows spatio-temporally resolved, full-field measurements rather than point-wise measurements. Furthermore, information on the crack width can be obtained simultaneously. To validate the experimentation, the authors processed image sequences of tests on four compact-tension specimens performed on a split-Hopkinson tension bar. The images were taken by a high-speed camera at a frame rate of 160,000 images per second. By applying the developed image sequence processing procedure to these datasets, crack propagation velocities of about 800 m/s were determined with a precision in the order of 50 m/s.

## 1. Introduction

To understand and describe the structural behavior of concrete, in-depth knowledge of the damage mechanisms is required. A crack formed in concrete is the result of a damage process, which starts with the formation and merging of micro-cracks. The evaluation of the resulting crack propagation velocity can provide valuable information on the process and rate of the damage. Such information is crucial to explaining the behavior of concrete at high loading rates. Several researchers have tried to explain the rate-dependent behavior of concrete based on the combined effects of high loading rate and low crack propagation velocity [1,2,3]. Given that crack formation does not occur instantaneously, but rather requires a certain amount of time for the development of damage, it can be deduced that under high loading rates, a strength-enhancing effect can emerge. Thus, a partial cause of the so-called strain rate effect [4,5,6,7] is likely attributable to the limited crack propagation velocity. The theoretical maximum of crack propagation velocity is the Rayleigh or surface wave velocity, that is the velocity at which energy can be transported along a surface. In concrete, the Rayleigh wave propagation velocity is approximately 2200 m/s [8]. However, experimental investigations by various researchers have been able to prove only significantly lower crack propagation velocities.

Bhargava/Rehnström [9] estimated crack propagation velocities of about 180 m/s based on film recordings taken in blast tests on concrete prisms. Mindess and Bentur resp. Mindess [10,11] also used camera images to evaluate impact-loaded beams and determined an approximate crack propagation velocity of about 100 m/s by comparing the crack progress interactively, frame-to-frame. Curbach [12] used notched concrete specimens with applied conductive lacquer barriers, which were destroyed one after the other depending on the crack propagation progress. Based on the measurement data, he postulated an upper limit of the crack velocity of about 500 m/s in concrete. Zhang et al. [13] investigated the crack velocity in high-strength concretes under different loading rates using strain gauges. They observed an increase in crack propagation velocity with the increasing loading rate. At low loading rates (10^−4^ to 10^1^ mm/s), significant differences in the velocity were found during cracking. Late-stage velocity was observed to be one order of magnitude higher when compared to that observed in earlier stages. However, under high loading rates (10^2^ to 10^3^ mm/s), a steadier crack propagation velocity was observed, with velocities in the range of 200 to 400 m/s. Acoustic emission [14] and digital image correlation (DIC) [15] can be considered as the most recent methods for measuring crack propagation speed. Obtaining the temporal position of the crack tip using high-speed camera shots in combination with displacement field analysis using a commercial DIC software [16] shows promise in the application of such novel methods. A crack propagation velocity of about 1300 m/s was obtained in spalling experiments on notched concrete specimens.

An obvious advantage of high-speed, camera-based techniques in detecting cracks and determining their crack propagation velocity is the fact that the entire surface of a sample can be analyzed simultaneously, thus enabling in turn the simultaneous observation of multiple cracks. However, interactive image sequence processing is rather tedious. Hence, automated techniques are required for the efficient processing of the experimental data. Reliable and precise image processing routines should also exclude observer errors and thus contribute to increasing the reproducibility of results. The article at hand presents an approach to crack velocity measurement based on automated high-speed camera image processing. A high-speed camera was used to monitor dynamic tests on compact-tension specimens at a frame rate of 160,000 images per second. The images were processed using an automated crack-detection and measurement procedure able to detect cracks with a width on the order of 0.1 pixel. In the following, the image sequence processing chain for crack detection is outlined. Then, the detection of crack tips (and their propagation velocities) from the image processing results are described. Finally, the results of applying the methods developed for high-speed camera image processing will be compared to those obtained using conventional conductive lacquer barrier measurements.

## 2. Photogrammetric Techniques for Crack Detection and Crack Width Measurement in High-Speed Camera Image Sequences

The image sequence processing chain, which is used for crack detection and crack measurement in the work presented here, bears the name “cascaded image analysis” in ref. [17]. It has been described in detail by Liebold and Maas [18,19,20] and is briefly summarized here. We limit ourselves to the analysis of specimens with planar surfaces as recorded by monocular image sequences.

**Figure 1:**
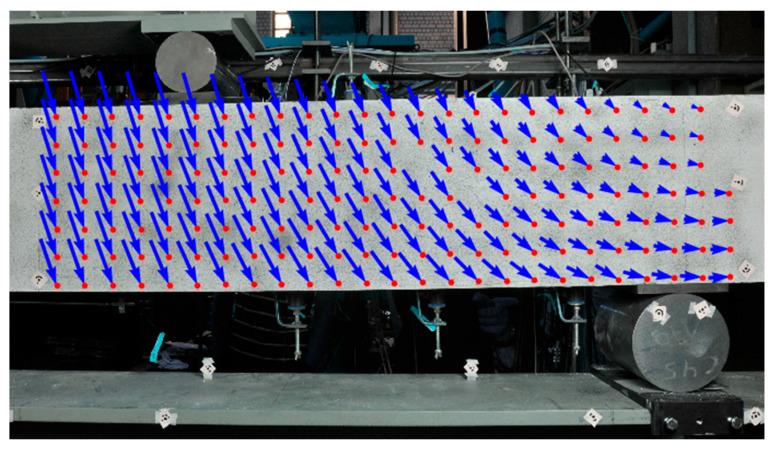
Displacement field, exaggerated using a vector scale factor of 6, from a bend test.

As a first step, a grid of points is defined in the camera image of the first epoch under zero load. The grid points are tracked through the consecutive images of the sequence using a sub-pixel accuracy least-squares image matching technique [21] in order to obtain displacement fields for each epoch with regard to the first epoch. Figure 1 shows the displacement field from a bend test on a concrete beam with a thinned-out point set, wherein cracks cause discontinuities in the displacement vector field.

**Figure 2:**
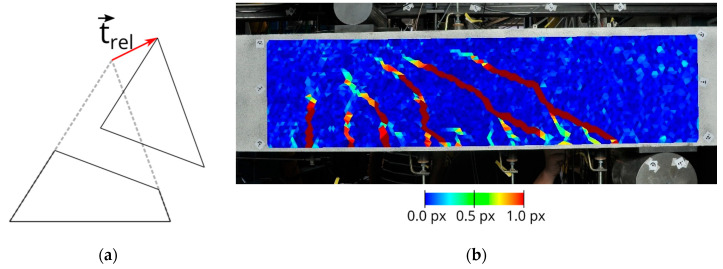
(**a**) Relative translation vector of a divided triangle; (**b**) color-coded map of the relative translation vector length $\left|\left|{\vec{t}}_{rel}\right|\right|$ for each triangle.

As an alternative to defining a regular grid of points to be tracked through the image sequence, an interest operator may be used to detect surface points with good image contrast, thus guaranteeing precise least-squares matching results. These points, which are not distributed in perfect regularity, are then triangulated into a mesh. The triangles of all images after the zero-load epoch are subsequently analyzed for deformations. Triangles with cracks penetrating them entirely show significant deformations, while the remaining triangles are unchanged. For that purpose, the relative translation vector ${\vec{t}}_{rel}$ can be computed for each triangle based on the relative movement of its vertices [19,20]. Figure 2a schematically depicts a divided triangle used as the assumed model for the computation of the relative translation vector.

**Figure 3:**
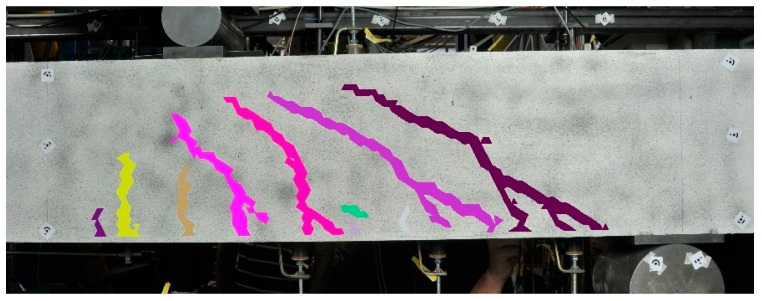
Connected components of crack triangles where $\left|\left|{\vec{t}}_{rel}\right|\right|>\delta$ according to [19].

The norm of this vector $\left|\left|{\vec{t}}_{rel}\right|\right|$ can be used as a deformation quantity for brittle material. Figure 2b shows a color-coded map of this quantity. The norm is checked for exceeding a threshold value δ, that is on an order of magnitude of the precision of the displacement field, i.e., approximately a tenth of a pixel. Due to the development of some distributional noise in the deformation field, filter methods such as the bilateral filter technique can be applied [18]. In classifying crack regions, the connected components of triangles where $\left|\left|{\vec{t}}_{rel}\right|\right|>\delta$ are determined by applying a region-growing technique including the hysteresis method with a second threshold, as performed by Canny [22]. Figure 3 shows an example of the connected components of deformed triangles from a load test with a concrete beam.

**Figure 4:**
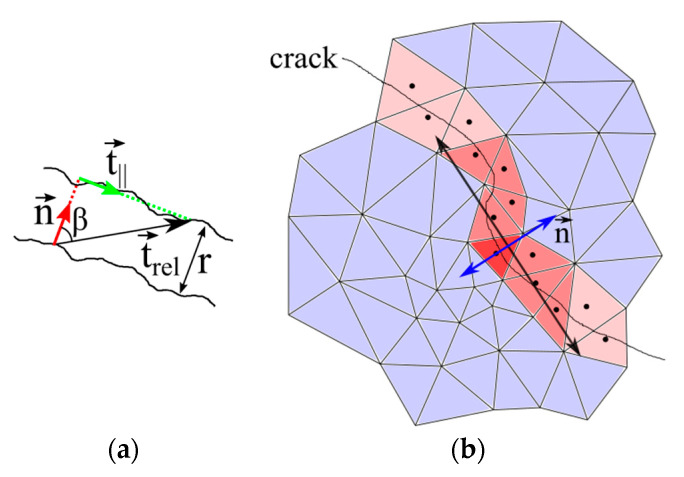
(**a**) The crack width r is computed by the projection of ${\vec{t}}_{rel}$ onto the normal $\vec{n}$; (**b**) crack normal estimation according to [19,20].

Furthermore, it is also possible to derive crack widths and crack opening vectors for deformed crack triangles with the help of the relative translation vector ${\vec{t}}_{rel}$ as shown by Liebold and Maas [19,20]. Crack widths can be computed as the projection of the relative translation vector ${\vec{t}}_{rel}$ onto the crack normal $\vec{n}$, which has to be determined first; see Figure 4a. For each deformed triangle, the crack normal can be estimated by a line fitted through neighboring deformed triangles; see Figure 4b.

The result of the image sequence processing chain described is a complete crack pattern for each image of the sequence plus subpixel accuracy information on local crack width. If a crack propagates, the crack pattern on the specimen’s surface and the measured crack widths will change. In the next step, these changes are analyzed to quantify the crack tip motion.

It is also possible to use and extend the algorithms presented here to measurements with stereo camera systems in order to analyze non-planar surfaces as shown by Liebold et al. [23,24]. 

## 3. Crack Tip Detection in Multi-Temporal Crack Pattern Images

**Figure 5:**
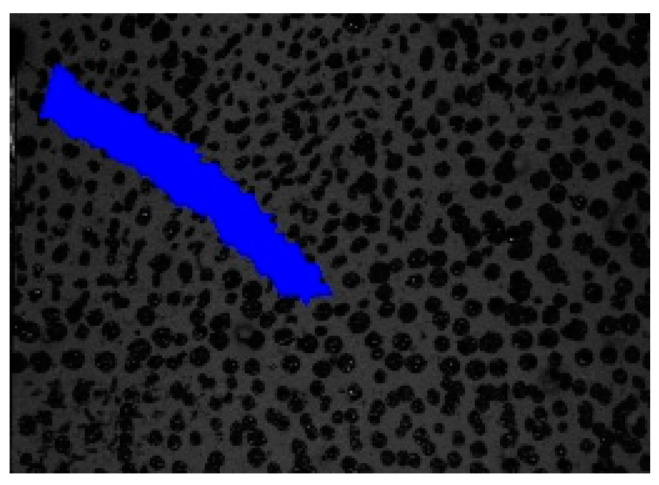
Connected components (in blue) of crack triangles.

To estimate the propagation velocity of cracks in an image sequence, it is necessary to detect the crack tips in each image. Direct detection of the crack tip in the images is restricted by the subpixel width of a crack near its tip. As a more reliable alternative, the crack tip position and motion can be determined indirectly using the algorithms presented in the previous section. Accordingly, the displacement field and the relative translation vectors are computed for each triangle of the mesh. Crack candidates are found by thresholding where $\left|\left|{\vec{t}}_{rel}\right|\right|>\;\delta_{1}$, and connected components are determined with a region-growing technique using the hysteresis method with a second threshold (δ_2_ = 0.5 × δ_1_); see Figure 5.

In the following, the crack tip must be found in the connected component of the deformed triangles. Two possible methods are presented here, an approach based on triangle neighborhood analysis and a principal component analysis approach. 

**Figure 6:**
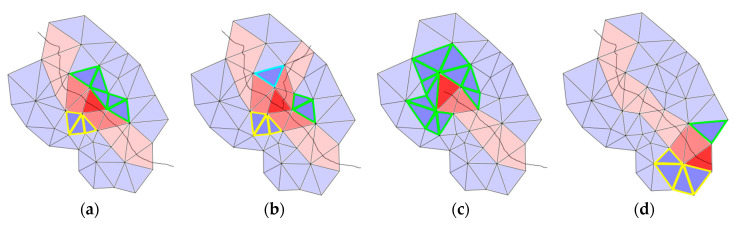
Neighborhood analysis: The bold red triangle is the crack candidate that is considered; darker blue triangles are non-deformed neighbor triangles: (**a**) Standard case with two components; (**b**) crack junction; (**c**) crack tip; (**d**) crack candidate at the mesh boundary.

The former method considers the neighborhood of each crack triangle. Figure 6 shows four possible cases in the neighborhood analysis of the crack candidates, shown as bold red triangles. In Figure 6a–c, connected components of non-deformed triangles, i.e., darker blue triangles with edges marked in thicker, colored lines, in the set of the neighbor triangles are determined. The number of these components are counted. Figure 6a shows the standard case with two components, Figure 6b represents a crack junction with more than two connected components around the dark red crack candidate (sets of triangles with yellow or green edge marking). In Figure 6c, only one component of non-deformed neighbor triangles is determined, implying a possible crack tip. Figure 6d shows a crack candidate at the boundary of the mesh, which can be analyzed with the help of the halfedge data structure [25]. A boundary triangle has at least one halfedge with no opposite halfedge partner.

**Figure 7:**
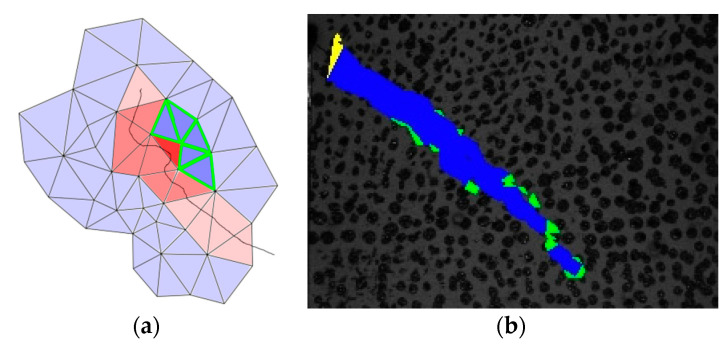
(**a**) False crack tip detection with the neighborhood analysis; (**b**) experimental example with false detections.

Sometimes, the classification of crack tips can fail as shown in Figure 7a. This might also be the case if the thickness of the area of deformed triangles is larger than one triangle, which can be caused by filtering methods, the smearing of $\left|\left|{\vec{t}}_{rel}\right|\right|$. Figure 7b depicts the connected component of crack triangles (blue) in an experiment. Mesh boundary triangles are colored yellow and triangles that are identified as crack tips are depicted in green.

**Figure 8:**
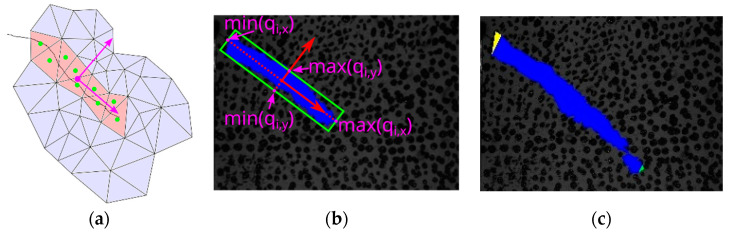
(**a**) Connected components of the deformed triangles in red, of which the center points are colored green, the mean and the principal directions magenta; (**b**) rotated bounding box computed using the principal component analysis (PCA); (**c**) crack tip detection by means of PCA. The yellow triangle is detected as a mesh boundary triangle, the green triangle shows the detected crack tip.

The latter method in crack tip identification is the principal component analysis (PCA), as shown in Figure 8. The set of center points of the deformed triangles from the connected component are analyzed by computing the mean and the covariance matrix; see Equations (1) and (2) as well as Figure 8a.

(1) $${\vec{x}}_{m}=\;\frac{1}{n}\;\cdot \overset{n}{\underset{i=1}{\sum }}{\vec{x}}_{i}$$

(2) $$\Sigma =\frac{1}{n-1}\cdot \overset{n}{\underset{i=1}{\sum }}({\vec{x}}_{i}-{\vec{x}}_{m})\cdot {\left({\vec{x}}_{i}-{\vec{x}}_{m}\right)}^{T}$$

where ${\vec{x}}_{i}$ = center of triangle *i* of the connected component, ${\vec{x}}_{m}$ = mean of the ${\vec{x}}_{i}$ values, *n* = number of triangles of the connected component, **Σ** = covariance matrix of the ${\vec{x}}_{i}$ values.

After this, the covariance matrix can be decomposed using an eigenvalue decomposition:

(3) $$\Sigma =V\cdot \Lambda \cdot V^{T}$$

where ***V*** = rotation matrix with the eigenvectors, ***Λ*** = eigenvalue matrix (diagonal).

The center points of the triangles of the connected components can then be transformed:

(4) $${\vec{q}}_{i}=V\cdot \left({\vec{x}}_{i}-\;{\vec{x}}_{m}\right)$$

The minimum and maximum values in the x and y directions belong to possible crack tip candidates. The dimension with the greater extension is chosen; see Equation (5) and Figure 8b.

(5) $$\mathrm{If}\;\left(\max\left(q_{i,x}\right)-\min\left(q_{i,x}\right)\right)>\left(\max\left(q_{i,y}\right)-\min\left(q_{i,y}\right)\right)\;i_{1}=argmin\left(q_{i,x}\right)\;\mathrm{and}\;i_{2}=argmax\left(q_{i,x}\right)\;\mathrm{otherwise}\;i_{1}=argmin\left(q_{i,y}\right)\;\mathrm{and}\;i_{2}=argmax\left(q_{i,y}\right)$$

Figure 8c illustrates the extension of the transformed coordinates of the triangle centers as a rotated bounding box. If the crack tip candidate is a mesh boundary triangle, the candidate should be rejected.

Due to possible movements of the entire specimen between the images, the crack tip position of the previous image should be transformed into the current time step. The correction can be done by adding the mean vertex displacement changes to the previous crack tip triangle center.

The PCA method works well for cracks without branches, but may fail in cases of branching. During the experimentation, the authors could observe only one crack without branches. Here, the PCA method produced fewer outliers than the first presented method and consequently was used.

## 4. Crack Velocity Determination

Crack velocities can be obtained as derivatives of the crack lengths over time. This is often done by numerical differentiation with, for instance, finite differences, i.e., forward and central differences. As this may lead to high fluctuations due to uncertainties, the authors decided to compute regression lines. The least-squares method offers the advantage of providing standard deviations as internal error measures. The derivatives are computed at each data point, including the four nearest neighbors. The mathematic model for the regression is:

*d* = *m* × *t* + *c* (6)

where *d* = crack length, *t* = time, *m* = velocity, *c* = *y*–intercept.

This can also be written in matrix notation:

(7) L+v=A·x

where ***A*** = design matrix, ***L*** = observation vector, ***x*** = vector of unknowns, **v** = residual vector.

For the i^th^ data point, the matrices are:

(8) $$L=\left(\begin{array}{c} d_{i-2}\\ d_{i-1}\\ d_{i}\\ d_{i+1}\\ d_{i+2} \end{array}\right)$$

(9) $$A=\left(\begin{array}{cc} 1 & t_{i-2}\\ 1 & t_{i-1}\\ 1 & t_{i}\\ 1 & t_{i+1}\\ 1 & t_{i+2} \end{array}\right)$$

(10) $$x=\left(\begin{array}{c} c\\ m \end{array}\right)$$

The measurements are weighted using binomial weights (1, 4, 6, 4, 1). The parameter vector **x** can be obtained by minimizing the residuals and solving the normal equations:

(11) $$A^{T}\cdot P\cdot A\cdot x=A^{T}\cdot P\cdot L$$

where ***P*** = weight matrix.

(12) $$P=\;\left(\begin{array}{ccccc} 1 & 0 & 0 & 0 & 0\\ 0 & 4 & 0 & 0 & 0\\ 0 & 0 & 6 & 0 & 0\\ 0 & 0 & 0 & 4 & 0\\ 0 & 0 & 0 & 0 & 1 \end{array}\right)$$

Then, the residual vector ***v*** can be calculated:

(13) v=A·x−L

The square of the *a posteriori* standard deviation $\sigma_{0}$ of the unit weight can be computed as follows:

(14) $$\sigma_{0}^{2}=\frac{v^{T}\cdot P\cdot v}{n-u}=\frac{v^{T}\cdot P\cdot v}{3}$$

where *n* = number of observations, *u* = number of unknowns.

The covariance matrix $\Sigma_{xx}$ of the parameters can be computed as follows:

(15) $$\Sigma_{xx}=\sigma_{0}^{2}\cdot {\left(A^{T}\cdot P\cdot A\right)}^{-1}$$

In addition to the actual velocity, the standard deviation of the velocity can be derived from Equation (15):

(16) $$\sigma_{m}=\sqrt{\Sigma_{xx,22}}$$

## 5. Velocity and Error Estimation of the Lacquer Barriers

The velocity between two lacquer barriers is:

(17) $$m=\frac{\Delta l}{\Delta t}$$

where Δ*l* = length between the conductive lacquer barriers, Δ*t* = time difference.

It is not possible to compute a standard deviation using the least-squares method to evaluate the error of the velocity measurement. However, the error can be estimated using variance propagation. For uncorrelated measurements, the propagated variance is:

(18) $$\sigma_{m}^{2}={\left(\frac{\partial m}{\partial \Delta l}\right)}^{2}\cdot \sigma_{\Delta l}^{2}+{\left(\frac{\partial m}{\partial \Delta t}\right)}^{2}\cdot \sigma_{\Delta t}^{2}=\frac{1}{\Delta t^{2}}\cdot \sigma_{\Delta l}^{2}+\frac{s^{2}}{\Delta t^{4}}\cdot \sigma_{\Delta t}^{2}=\frac{1}{\Delta t^{2}}\cdot \left(\sigma_{\Delta l}^{2}+m^{2}\cdot \sigma_{\Delta t}^{2}\right)$$

To estimate the standard deviation $\sigma_{m}$, the standard deviations of the measurements $\sigma_{\Delta l}$ and $\sigma_{\Delta t}$ are required. The thickness of a conductive lacquer barrier is about 3 mm and the expected velocity is about 800 m/s such that a crack needs about 4 µs to traverse it. It is not certain at which position the electrical contact is interrupted. Two barriers must be passed and the angle of impact is not necessarily 90°. Based on these considerations, we assume roughly $\sigma_{\Delta t}$ = 5 µs and $\sigma_{\Delta l}$ = 3 mm. The standard deviation $\sigma_{m}$ increases with decreasing Δt.

## 6. Experimental Program

### 6.1. Material under Investigation

A normal-strength matrix containing cement, fly ash, and fine sand were used to produce the compact-tension specimens. Table 1 provides the mixture compositions. The finely grained matrix is used as the constituent matrix of strain hardening cement-based composites (SHCC), and its behavior under impact tensile loading has been investigated previously. Since the matrix was named M1 in the previous investigations [26,27], the same name is used in this study.

### 6.2. Dynamic Test Setup

**Figure 9:**
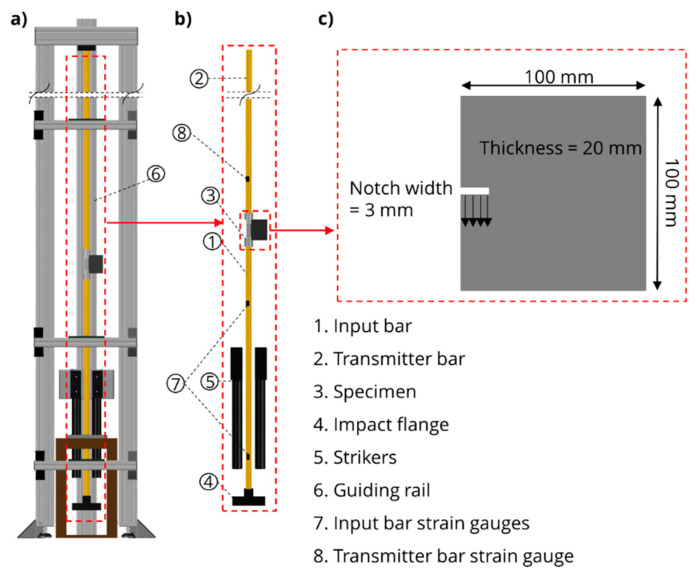
Experimental setup: Split-Hopkinson tension bar used for performing dynamic tests on compact-tension specimens: (**a**) Schematic view of the setup; (**b**) main components of the assembly; (**c**) specimen and its dimensions.

**Figure 10:**
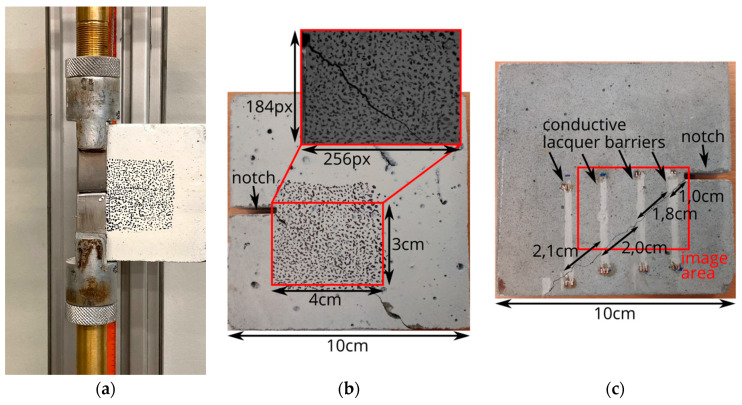
(**a**) Specimen in the test setup. (**b**) Front side of the specimen; (**c**) back side with four conductive lacquer barriers.

The dynamic tests on compact-tension specimens were performed in a split-Hopkinson tension bar (SHTB). Figure 9 provides the schematic view of the setup and geometry of the specimens. The SHTB’s input and transmitter bars, both with a diameter of 24 mm, are made of brass with a Young’s modulus of 98 GPa. Adapters were used to fix the compact-tension specimens in the SHTB. The loading principle in the SHTB, based on the propagation of an elastic wave in the input bar, was used to achieve a high displacement rate in compact-tension specimens. A detailed description of the SHTB can be found in ref. [28]. Figure 10a shows the specimen in the test setup.

A high-speed camera of type Photron Fastcam SA-X2 (produced by Photron Limited, Tokyo, Japan) was used to monitor the front surface of the specimen during the experiment. The frame rate of the camera was set to 160,000 fps at a resolution of 256 × 184 pixels which covered approximately a rectangle of 40 mm × 30 mm on the specimen’s surface; see Figure 10b. The recorded frames were subsequently used for performing the image sequence processing chain as outlined above. Additionally, four barriers made of conductive lacquer were placed on the rear surface of the specimens for validation as presented in Figure 10c. The conductive barriers were each connected a voltage supply to the signal acquisition device. An oscilloscope of type Pico 4824 produced by Pico Technology, St Neots, UK, was used to record the voltage signals at 20 MHz. Upon propagation of the crack through the barriers, voltage signals were reduced to zero so that the time needed for the crack to propagate between barriers can be obtained. In total, four compact-tension specimens made of M1 matrix were tested in the SHTB.

## 7. Experimental Results

**Figure 11:**
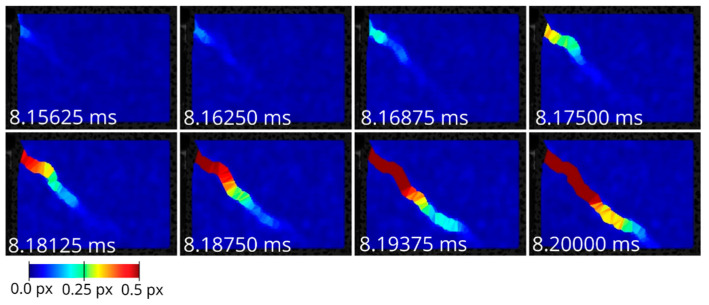
Color-coded maps of $\left|\left|{\vec{t}}_{rel}\right|\right|$ of a sequence of one of the experiments at the 160 kHz imaging rate, time stamps in milliseconds.

**Figure 12:**
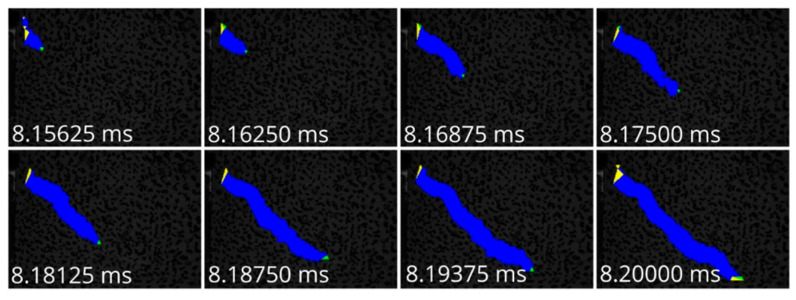
Crack triangles (blue) with crack tips, i.e., green colored triangles and the yellow-colored mesh border triangles for the experiment presented in Figure 11.

For the detection of the deformed triangles, the algorithm described in Section 3 and Section 4 was applied. Figure 11 shows color-coded maps of the relative translation vector lengths for each triangle for different time steps of one of the experiments. Then, the thresholding was done for the norm of the relative translation vector with different threshold values δ (0.08, 0.10, 0.12 px). The connected components and the crack tips were determined by means of the PCA method. Figure 12 shows an example with a threshold of 0.10 px.

**Figure 13:**
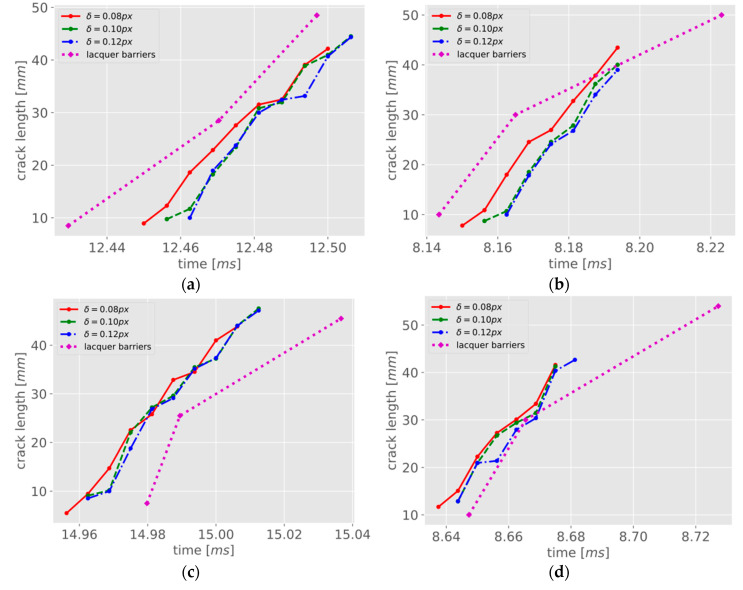
Crack length over time plots using the photogrammetric method with different thresholds for deformed triangles (red, green, blue) and using the conductive lacquer barriers (magenta): (**a**) 1st sample; (**b**) 2nd sample; (**c**) 3rd sample; (**d**) 4th sample.

The propagation length between the consecutive images, and accordingly its velocity, were determined automatically using the image processing chain described in the previous sections. The distance from the notch tip, that is the beginning of the crack, to the first crack tip was measured manually at the left border of the image since the notch tip was not included in the measuring field of the optical sensor. This measure is not necessary for the velocity determination but is useful for the comparison to the lacquer barriers in the crack length over the course of time. Figure 13 shows the crack propagation lengths as a function of time for the different thresholds δ for $\left|\left|{\vec{t}}_{rel}\right|\right|$ as well as for the first three conductive lacquer barriers for the four specimens tested. The last barrier was ignored due to crack branching which happened between the third and fourth barriers. In addition, the optical measurement range does not cover the last barrier; see also Figure 10b. As expected, the photogrammetric method with the threshold of δ = 0.08 px is the most sensitive, detecting crack tips earlier in the image sequence than if higher thresholds are used. The magenta-dotted line shows the crack length as a function of time for the conductive lacquer barriers. Comparing the methods, both techniques show monotonically increasing behavior of similar intensity. There is a slight mismatch in the time domain, which might be caused by synchronization errors but is not relevant to velocity determination.

**Figure 14:**
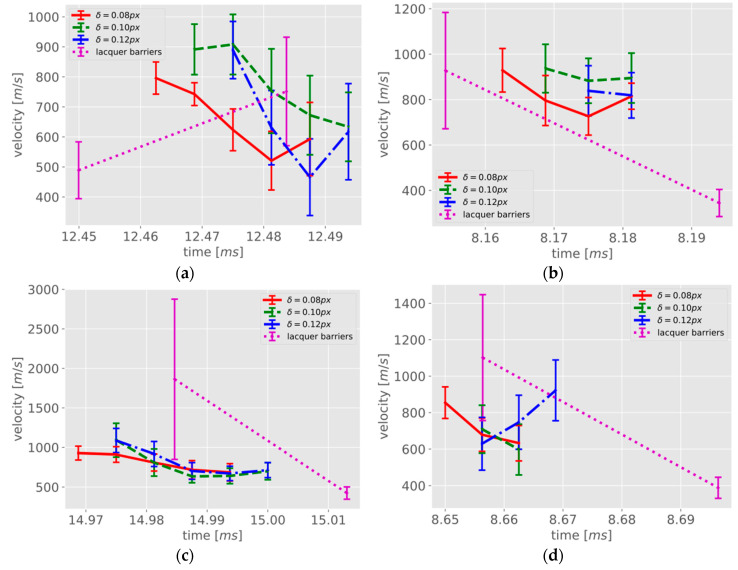
Crack propagation velocities and the corresponding error bars (±one standard deviation) obtained using the photogrammetric technique with different thresholds for deformed triangles (red, green, blue) and using the conductive lacquer barriers (magenta): (**a**) 1st sample; (**b**) 2nd sample; (**c**) 3rd sample; (**d**) 4th sample.

Next, the velocities were computed as derivatives of the crack lengths with respect to time. Figure 14 shows the results for the four specimens. The 5-point least-squares velocities obtained by the photogrammetric method for the different thresholds and the two velocities calculated between the conductive lacquer barriers are presented. In addition to the velocities, errors bars are plotted, showing the range of ± one standard deviation. The photogrammetric curves show some dependence on the threshold. This leads to an offset of the determined velocities on the time axis and to a certain fluctuation in the velocities as determined. In fact, as noted above, lower threshold values lead to an earlier detection of the crack tip. However, the resulting shift on the time axis is irrelevant to the velocity calculations. For the conductive lacquer barriers, only two velocity values can be derived. This is not sufficient for a comparison over the entire time span in which the photogrammetric method was used. However, the velocities are on the same order of magnitude. The standard deviations of the velocity values obtained with the photogrammetric method are in a range between 40 and 170 m/s, the standard deviations of the lacquer barrier based velocities reach several hundred m/s.

**Figure 15:**
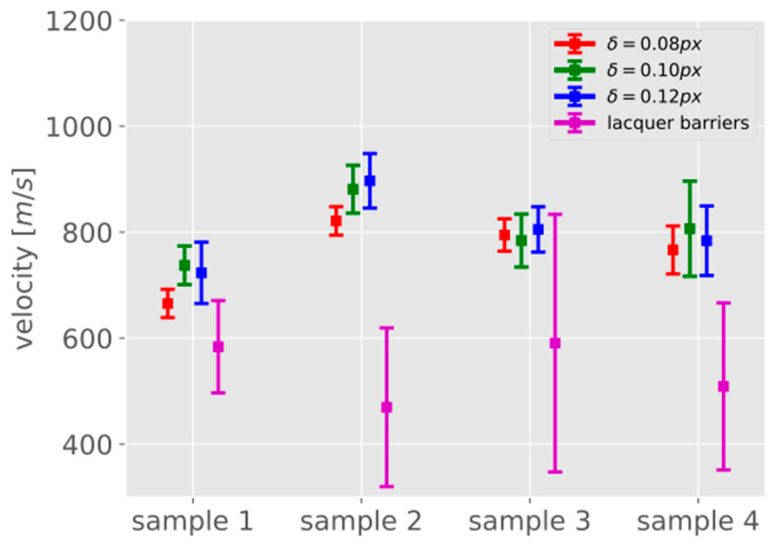
Least-squares velocities obtained using all data points as average velocities and the corresponding standard deviations (error bars: ±one standard deviation) for all specimens.

Due to the relatively high standard deviations, the image sequence based instantaneous velocities can also be translated into average velocities using the least-squares method for all data points. Figure 15 shows the corresponding results: The average velocities obtained with the photogrammetric techniques vary between 650 and 900 m/s with standard deviations in the range of 30 to 90 m/s. The velocities between the conductive lacquer barriers are lower, in a range between 490 and 600 m/s, with higher standard deviations of up to 240 m/s. It is obvious that the velocities obtained from the high-speed camera data are consistently higher than the values obtained from the conductive lacquer barriers. However, it should be noted also that the standard deviations of the lacquer barrier measurements are much higher, and thus less reliable as only three data points are used in the computation. The number of conductive lacquer barriers in the measuring area of the camera could be increased in order to have more data points and to compare the methods, but this may also lead to increasing measurement errors due to the small distances between the barriers. This indicates that the quality of the conductive lacquer barriers measurements cannot be considered as a reference for the results obtained from the photogrammetric image processing chain due to their lower quality.

## 8. Conclusions

The article at hand presents a novel technique for determining crack propagation velocity on the basis of recorded high-speed camera image sequences. A precise, automatic, photogrammetric image sequence processing chain was developed, implemented, and validated. While using a high-speed camera means a relatively high instrumental effort, it provides the obvious advantage of offering spatio-temporally resolved, non-contact measurements rather than single-point measurements. The technique is able to detect cracks with a width on the order of 0.1 pixel and may thus be applied even in tracking fissures not visible to the human eye. The crack propagation velocities obtained from four laboratory experiments conducted in this study were on the order of 800 m/s.

For the experiments, a camera with a frame rate of 160,000 fps was used. The high frame rate comes with the drawback of an image format limited to 256 × 184 pixels. Future high-speed cameras will offer higher frame rates and larger image formats, which will be beneficial both for the number of cracks to be analyzed simultaneously and for the precision of the velocity values obtained by the method developed.

## Figures and Tables

**Table 1:** Composition of the strain hardening cement-based composites (SHCC) matrix M1 under investigation.

M1 Matrix	(kg/m^3^)	Producer
CEM I 42.5R-HS	505	SCHWENK Zement KG, Ulm, Germany
Fly ash Steament H4	621	STEAG GmbH, Essen, Germany
Quartz sand 0.06 - 0.2 mm	536	Strobel Quarzsand GmbH, Freihung, Germany
Viscosity modifying agent	4.8	Sika, Sidney, Australia
Water	338	-
Superplasticizer Glenium ACE 30	10	BASF, Ludwigshafen, Germany

## References

[1] Lu Y., Li Q. (2011). About the dynamic uniaxial tensile strength of concrete-like materials. Int. J. Impact Eng..

[2] Rossi P., Toutlemonde F. (2011). Effect of loading rate on the tensile behaviour of concrete: Description of the physical mechanisms. Mater. Struct..

[3] Häussler-Combe U., Kühn T. (2012). Modeling of strain rate effects for concrete with viscoelasticity and retarded damage. Int. J. Impact Eng..

[4] Mosig O., Curbach M. (2020). The crack propagation velocity as a reason for the strain rate effect of concrete: An analytical model. Civ. Eng. Des..

[5] Abrams D.A. (1917). Effect of Rate of Application of Load on the Compressive Strength of Concrete. Proceedings, Part II. Am. Soc. Test. Mater..

[6] Evans R.H. (1942). Effect of Rate of Loading on the Mechanical Properties of some Materials. J. Inst. Civ. Eng..

[7] Bachmann H. (1993). Die Massenträgheit in einem Pseudo-Stoffgesetz für Beton bei schneller Zugbeanspruchung. Ph.D. Thesis.

[8] Reinhardt H.W., Weerheijm J. (1991). Tensile fracture of concrete at high loading rates taking account of inertia and crack velocity effects. Int. J. Fract..

[9] Bhargava J., Rehnström A. (1975). High-Speed Photography for Fracture Studies of Concrete. Cem. Concr. Res..

[10] Mindess S., Bentur A. (1985). A Preliminary Study of the Fracture of Concrete Beams under Impact Loading Using High Speed Photography. Cem. Concr. Res..

[11] Mindess S. (1995). Crack velocities in concrete subjected to impact loading. Can. J. Phys..

[12] Curbach M. (1987). Festigkeitssteigerung von Beton bei hohen Belastungsgeschwindigkeiten. Ph.D. Thesis.

[13] Zhang X.X., Yu R.C., Ruiz G., Tarifa M., Camara M.A. (2010). Effect of loading rate on crack velocities in HSC. Int. J. Impact Eng..

[14] Goszczynska B. (2014). Analysis of the process of crack initiation and evolution in concrete with acoustic emission testing. Arch. Civ. Mech. Eng..

[15] Bu J., Chen X., Hu L., Yang H., Liu S. (2020). Experimental Study on Crack Propagation of Concrete Under Various Loading Rates with Digital Image Correlation Method. Int. J. Concr. Struct. Mater..

[16] Forquin P. (2012). An optical correlation technique for characterizing the crack velocity in concrete. Eur. Phys. J. Spec. Top..

[17] Hampel U., Maas H.G. (2009). Cascaded image analysis for dynamic crack detection in material testing. ISPRS J. Photogramm. Remote. Sens..

[18] Liebold F., Maas H.G. (2016). Advanced spatio-temporal filtering techniques for photogrammetric image sequence analysis in civil engineering material testing. ISPRS J. Photogramm. Remote. Sens..

[19] Liebold F., Maas H.G. (2018). Sub-pixel accuracy crack width determination on concrete beams in load tests by triangle mesh geometry analysis. ISPRS Ann. Photogramm. Remote. Sens. Spat. Inf. Sci..

[20] Liebold F., Maas H.G. (2020). Strategy for Crack Width Measurement of Multiple Crack Patterns in Civil Engineering Material Testing using a Monocular Image Sequence Analysis. PFG–J. Photogramm. Remote. Sens. Geoinf. Sci..

[21] Ackermann F. (1984). Digital Image Correlation: Performance and Potential Application in Photogrammetry. Photogramm. Rec..

[22] Canny J. (1986). A Computational Approach to Edge Detection. IEEE Trans. Pattern. Anal. Mach. Intell..

[23] Liebold F., Maas H.G., Heravi A.A. (2019). Crack Width Measurement for Non-planar Surfaces by Triangle Mesh Analysis in Civil Engineering Material Testing. Int. Arch. Photogramm. Remote. Sens. Spat. Inf. Sci..

[24] Liebold F., Maas H.G., Deutsch J. (2020). Photogrammetric determination of 3D crack opening vectors from 3D displacement fields. ISPRS J. Photogramm. Remote. Sens..

[25] Botsch M., Knobbelt L., Pauly M., Alliez P., Levy B. (2010). Polygon Mesh Processing.

[26] Curosu I., Mechtcherine V., Forni D., Cadoni E. (2017). Performance of various strain-hardening cement-based composites (SHCC) subject to uniaxial impact tensile loading. Cem. Concr. Res..

[27] Heravi A.A., Curosu I., Mechtcherine V. (2020). A gravity-driven split Hopkinson tension bar for investigating quasi-ductile and strain-hardening cement-based composites under tensile impact loading. Cem. Concr. Compos..

[28] Heravi A.A., Fuchs A., Gong T., Curosu I., Kaliske M., Mechtcherine V. (2020). Mechanical characterization of textile reinforced cementitious composites under impact tensile loading using the split Hopkinson tension bar. Cem. Concr. Compos..

